# Peritoneal Sarcoidosis Mimicking Peritoneal Tuberculosis and Advanced Ovarian Carcinoma

**DOI:** 10.1155/2020/1905649

**Published:** 2020-07-06

**Authors:** Brittany N. Robles, Catherine Shea, Ghadir Salame

**Affiliations:** Obstetrics and Gynecology, Wyckoff Heights Medical Center, Brooklyn 11237, USA

## Abstract

Sarcoidosis is an inflammatory disease that affects one or multiple organs, most commonly the lungs and lymph nodes. This disease can present in a variety of ways which often makes diagnosis difficult. A 54-year-old postmenopausal African American female with a history of omental carcinomatosis of unknown origin was referred to the gynecology-oncology service at a local community hospital following a laparoscopic incarcerated hernia repair where multiple abdominal lesions suspicious of ovarian carcinomatosis were visualized. She was brought to the operating room for a diagnostic laparoscopy at which point the intra-abdominal survey revealed white tubercle-like lesions that were consistent with peritoneal tuberculosis. The lesions were excised and sent to pathology. The omentum biopsy was originally reported as adipose tissue showing focal fibrosis, focal mild acute inflammation, few cyst formation, and multiple granulomatous chronic inflammation, with multinucleated giant cells. Periodic acid-Schiff stain and acid fast bacilli stain were negative, and a diagnosis of peritoneal tuberculosis was made. The patient was started on an antituberculosis treatment regimen; however, she was not improving. The pathology slides were reexamined and revealed nonnecrotizing granulomatous inflammation consistent with sarcoidosis. The patient was immediately referred to the department of pulmonology and rheumatology, at which point she was started on corticosteroids and had an improvement in her condition.

## 1. Introduction

Sarcoidosis is a systemic disease of unknown etiology with a wide variety of clinical and radiological manifestations [1]. It affects 1 to 6 of every 1000 people, worldwide, with an incidence of approximately 1 in 10,000 in the United States [2]. African Americans are at a 3- to 20-fold higher rate compared to whites, with women 10 times more likely to be affected than men [3, 4]. Sarcoidosis is most commonly seen in the pulmonary and lymphatic system but can be seen in other organs which makes diagnosis very difficult [5, 6]. Peritoneal involvement frequently occurs, presenting as ascites, peritoneal thickening and multiple soft tissue nodules, mimicking peritoneal carcinomatosis [7].

Sarcoidosis affects males and females of all ages and races; however, it is most prevalent in African American, Afro-Caribbean, Swedish, and Danish individuals due to a genetic predisposition [8]. It is rarely reported in individuals of Spanish, Portuguese, Indian, Saudi Arabian, or South American descent possibly due to the lack of mass screening programs, and the predominance of other common granulomatous diseases such as tuberculosis, fungal infections, and Hansen's disease [9].

Sarcoidosis is typically diagnosed in women between the ages of 20 and 40 years of age with African American women being at greatest risk [5]. In addition, African American women tend to experience a more severe, long-term form of the disease, mainly affecting the lungs. Interestingly, women tend to have extrapulmonary involvement, seen in approximately 30% of patients, with the abdomen being the most frequent site [10, 11]. More than 1/3 of patients with sarcoidosis can develop chronic, unremitting inflammation with progressive organ impairment [12].

Diagnosing sarcoidosis mainly depends on clinical and radiological findings as well as the pathologic evidence of noncaseating epitheloid cell granulomas with the exclusion of other granulomatous diseases [13]. Given the high rate of spontaneous resolution, the decision of initiating immunosuppressive treatment must be carefully evaluated [14].

## 2. Case Presentation

A 54-year-old postmenopausal African American female, G4P3013, with a past medical history of type 2 diabetes mellitus, hypertension, hypothyroidism, and stage 5 chronic kidney disease with multiple hospitalizations for hypercalcemia as well as recurrent pleural effusions, with a history of omental carcinomatosis of unknown origin, was referred to the gynecology-oncology service at a local community hospital following a laparoscopic incarcerated hernia repair where multiple abdominal lesions suspicious of ovarian carcinomatosis were visualized. The lesions were noted throughout the abdomen including the abdominal wall, bowel, liver, and pelvis. Biopsies of the lesions taken from the omentum and abdominal wall were found to be granulomatous with chronic inflammation and multinucleated giant cells. Some granulomas were matted, and others showed foreign bodies containing crystalloid material and rare granulomatous necrosis. Periodic acid-Schiff stain and acid fast bacilli stain were negative. Although cytology did not suggest neoplasm, the patient was immediately referred to the gynecology-oncology clinic due to the suspicion of an ovarian neoplasm.

On presentation to gynecology-oncology clinic, the patient offered no gynecological complaints but endorsed nonspecific symptoms. She complained of anorexia and accompanied weight loss. Hospital records confirmed a fifty-nine-pound weight loss over seven months. She also complained of a chronic cough with scant-sputum production. She denied abdominal pain, abdominal cramping, vaginal bleeding, abdominal bloating, nausea, vomiting, and constipation. On exam, the patient was noted to have abdominal ascites, and her CA-125 level was elevated at 57.4 U/ml, which had risen from 39.9 U/ml a month prior. Following the appointment, she was scheduled for a diagnostic laparoscopy with possible hysterectomy and bilateral salpingo-oophorectomy; however, surgery was delayed due to her being hospitalized for symptomatic hypercalcemia and recurrent pleural effusions.

Once the patient was stabilized, she was taken to the operating room for diagnostic laparoscopy. Intra-abdominal survey revealed white tubercle-like lesions that were consistent with peritoneal tuberculosis. Laparoscopic scissors were used to excise a piece of the peritoneal wall containing the tubercle-like lesions. The tissue was then sent to pathology.

The omental biopsy was originally reported as adipose tissue showing focal fibrosis, focal mild acute inflammation, few cyst formation, and multiple granulomatous chronic inflammation, with multinucleated giant cells. Periodic acid-Schiff stain and acid fast bacilli stain were negative, and a diagnosis of peritoneal tuberculosis was made. The patient was immediately started on rifampin, isoniazid, pyrazinamide, ethambutol (RIPE) therapy, supplemented with azithromycin and vitamin B_6_.

**Figure 1:**
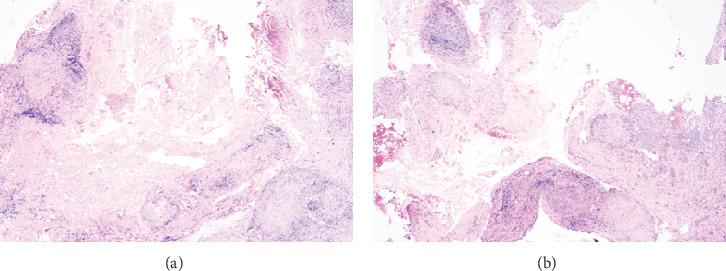
(a, b) Low power view (4x) of the peritoneal biopsy. Fat, few skeletal muscles, and nonnecrotizing granulomas, some are confluent.

**Figure 2:**
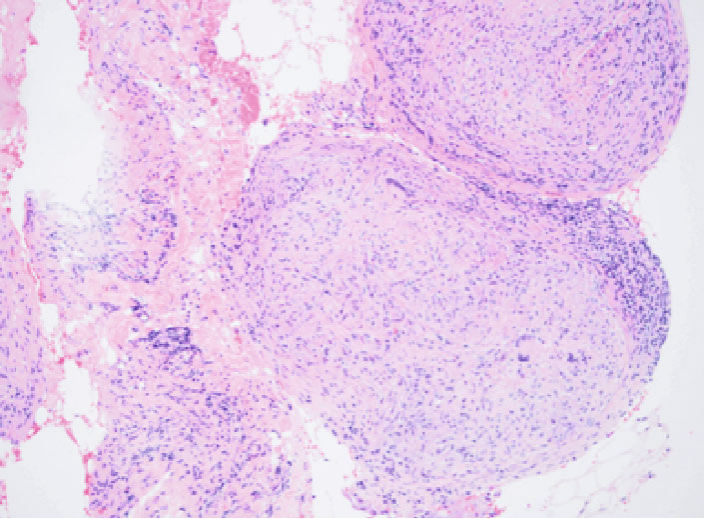
Medium power (10x) showing granuloma composed of epithelioid histiocytes and multinucleated giant cells.

**Figure 3:**
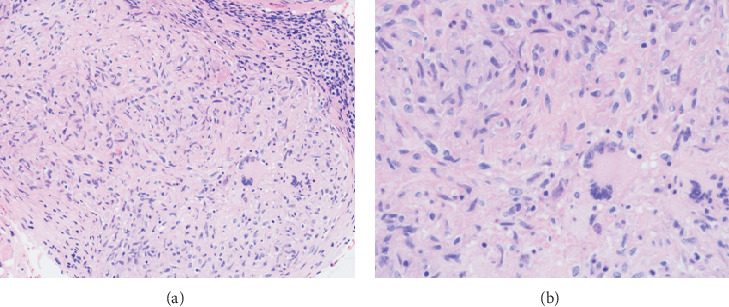
(a, b) High power view of the epithelioid histiocytes and multinucleated giant cells.

After several weeks of RIPE therapy, the patient was not showing any signs or improvement. Adenosine deaminase level was tested in the patients' pleural fluid and found to be elevated at 28.7 U/l. She also had a quantiferon test performed which was indeterminate. Decision was made to have the pathology from the tissue sample taken during surgery reviewed. The pathology revealed nonnecrotizing granulomatous inflammation (Figures 1(a) and 1(b)) and multinucleated giant cells (Figures 2 and 3(b)) consistent with sarcoidosis. The patient was immediately referred to the department of pulmonology and rheumatology, at which point she was started on corticosteroids and had an improvement in her condition.

## 3. Discussion

Correct identification and differentiation of peritoneal sarcoidosis from ovarian carcinoma and peritoneal tuberculosis in middle-aged females continues to be a significant challenge to the medical community. According to the literature, gastrointestinal involvement of sarcoidosis is very rare, seen in approximately 1% of all cases [15], with sarcoidosis of the serosal surface even scarcer [15]. Women with sarcoidosis are most often affected between the second and fourth decade of life [15], and clinically, they may present with pleural effusion, abdominal pain, ascites, vomiting, anorexia, hiccups, and even intestinal obstruction [16–19].

Sarcoidosis has been referred to by some experts as a partial autoimmune disease [20, 21]. Granuloma development in sarcoidosis is due to the deposition of y-globulins in response to an immune stimulus [22]. This stimulus is explained by a defect in the suppressor T cells, which allows the release of a “non-self” clone that causes an organ-specific reaction. This reaction then causes an abnormal activation of B cells which increases serum IgG, IgM, and IgA levels by 50%, 25%, and 10%, respectively [22].

However, nonspecific symptoms such as abdominal pain, bloating, and weight loss, coupled with an elevated CA-125, should raise concern for peritoneal sarcoidosis. Carbohydrate antigen or cancer antigen 125 (CA 125) is a tumor marker that is secreted by the mesothelial cells lining the peritoneal cavity as well as many other tissues of nonmesothelial origin such as tracheobronchial epithelium, epithelium of the pancreas, colon, gallbladder, stomach, kidney, breast, amniotic tissue, placenta, and cervical mucus membrane, making it a nonspecific tumor marker [1]. Furthermore, illnesses such as endometriosis and pelvic inflammatory disease can further increase the level of CA-125 as well as menstruation, pregnancy, and surgery such as laparotomy [23].

Nonnecrotizing granulomas are a characteristic pathologic finding of sarcoidosis. Caseation is absent in these granulomas but occasionally fibrinoid necrosis may be seen [24]. Fibrinoid necrosis is prominent in areas where several granulomas have coalesced and may be distinguished from caseation by the presence of a fine reticulin pattern on silver staining [25]. Epithelioid cells within sarcoid granulomas are thought to produce angiotensin-converting enzyme (ACE), resulting in elevated serum ACE levels in approximately 60% of patients with sarcoidosis [26]. ACE levels are thought to be proportional to the total granuloma volume, indicative of sarcoidosis activity [26]. In one population-based cohort study, the sensitivity and specificity of an elevated ACE level for diagnosing sarcoidosis were 41.4% and 89.9%, respectively [27]. Due to the small size of the granulomatous nodules, they often go undetected on CT scan [18, 19]; hence, laparoscopy and/or laparotomy is almost always essential to demonstrate the involvement of the disease. During laparoscopy and/or laparotomy, there may be evidence of ascites, diffuse thickening of the peritoneum, peritoneal nodules, and possibly an omental cake appearance. All of these findings are consistent with both peritoneal carcinomatosis and tuberculous peritonitis [26]; however, peritoneal biopsies are crucial for the diagnosis of sarcoidosis [13].

Although an isolated occurrence of sarcoidosis in the genital system is rare, if disease does occur here, the uterus and ovaries are the most common sites affected, most often in reproductive aged women [28, 29]. Symptoms of amenorrhea, menorrhagia, meto-menorrhagia, and postmenopausal bleeding can be experienced [30]. Women may also have symptoms of sarcoidosis due to cervical erosions, endometrial polypoid lesions, and recurrent serometra that can occur due to the disease course [30]. Like uterine sarcoidosis, most ovarian sarcoidosis that has been reported has been seen during the reproductive ages with a few cases seen in postmenopausal women [31].

Serosal involvement of sarcoidosis is rarely reported, especially due to the unique manifestation of this disease [32]. Moreover, the serum level of CA-125 may be elevated leading to the confusion and misdiagnosis of ovarian carcinoma [33, 34]. Many cases of peritoneal sarcoidosis proceed with a benign course, resolving spontaneously, in 33% of patient [33] or with a short course of corticosteroids [35] and in some cases, the addition of methotrexate [35]. Advanced sarcoidosis is associated with significant morbidity and mortality seen in approximately 25% of all patients [36].

Elevated CA-125 in addition to nonspecific symptoms such as fatigue, weight loss, dry cough, fever, night sweats, and shortness of breath should raise suspicion for peritoneal sarcoidosis. Women with peritoneal sarcoidosis who are misdiagnosed as having ovarian carcinoma often undergo unnecessary debulking operations which is the treatment of choice for malignant ovarian carcinoma. Radical treatment of this neoplasm generally involves removal of the uterus, fallopian tubes, ovaries, omentum, pelvic, and retroperitoneal lymph nodes, as well as debulking for metastatic spread. Misdiagnosing peritoneal sarcoidosis as an ovarian neoplasm can thus result in increased morbidity and mortality for young women undergoing such invasive surgical interventions.
